# Preclinical Validation of a Novel Injection-Molded Swab for the Molecular Assay Detection of SARS-CoV-2

**DOI:** 10.3390/diagnostics12010206

**Published:** 2022-01-15

**Authors:** Chiara E. Ghezzi, Devon R. Hartigan, Justin P. Hardick, Rebecca Gore, Miryam Adelfio, Anyelo R. Diaz, Pamela D. McGuinness, Matthew L. Robinson, Bryan O. Buchholz, Yukari C. Manabe

**Affiliations:** 1 Biomedical Engineering Department, University of Massachusetts Lowell, Lowell, MA 01854, USA; Devon_Hartigan@student.uml.edu (D.R.H.); Rebecca_Gore@uml.edu (R.G.); miryam_adelfio@student.uml.edu (M.A.); anyelo_diaz@student.uml.edu (A.R.D.); Pamela_McGuinness@uml.edu (P.D.M.); bryan_buchholz@uml.edu (B.O.B.); 2 Division of Infectious Diseases, Department of Medicine, John Hopkins University School of Medicine, Baltimore, MD 21287, USA; jhardic1@jhmi.edu (J.P.H.); mrobin85@jhmi.edu (M.L.R.); ymanabe@jhmi.edu (Y.C.M.)

**Keywords:** COVID-19, SARS-CoV-2, virological testing, diagnostic testing, swab, tissue model

## Abstract

During the COVID-19 public health emergency, many actions have been undertaken to help ensure that patients and health care providers have timely and continued access to high-quality medical devices to respond effectively. The development and validation of new testing supplies and equipment, including collection swabs, has helped to expand the availability and capability for various diagnostic, therapeutic, and protective medical devices in high demand during the COVID-19 emergency. Here, we report the initial validation of a new injection-molded anterior nasal swab, ClearTip™, that was experimentally validated in a laboratory setting as well as in independent clinical studies in comparison to gold standard flocked swabs. We have also developed an in vitro anterior nasal tissue model which offers a novel, efficient, and clinically relevant validation tool to replicate the clinical swabbing workflow with high fidelity, while being accessible, safe, reproducible, and time- and cost-effective. ClearTip™ displayed greater inactivated virus release in the benchtop model, confirmed by its greater ability to report positive samples in a small clinical study in comparison to flocked swabs. We also quantified the detection of biological materials, as a proxy for viral material, in multi-center pre-clinical and clinical studies which showed a statistically significant difference in one study and a reduction in performance in comparison to flocked swabs. Taken together, these results emphasize the compelling benefits of non-absorbent injection-molded anterior nasal swabs for COVID-19 detection, comparable to standard flocked swabs. Injection-molded swabs, as ClearTip™, could have the potential to support future swab shortages, due to its manufacturing advantages, while offering benefits in comparison to highly absorbent swabs in terms of comfort, limited volume collection, and potential multiple usage.

## 1. Introduction

The emergence of SARS-CoV-2 and the subsequent COVID-19 pandemic has resulted in over 200 million cases globally [1]. The long incubation period, as well as the high prevalence of asymptomatic carrier transmission, are prominent factors for the high transmission rate of SARS-CoV-2 [2]. Thus, the efficient and reliable detection of infectious carriers through large-scale surveillance is critical to limit further spreading of the disease, particularly as many countries experience multiple waves of infections. Despite effective vaccines, the continued global propagation of variants with efficient transmission dynamics even in vaccinated hosts increases the need to provide easily accessible testing on a wide variety of platforms.

A nasopharyngeal (NP) swab is the gold standard sampling method to detect SARS-CoV-2, as recommended by the World Health Organization (WHO) [3]. Sample collection with an NP swab is performed by trained professionals, transferred into transport media, and then processed via RNA purification and RT-qPCR, or other FDA-approved methods. NP swabs are 15 cm long to reach the posterior nasopharynx, with a head diameter between 1 and 3.2 mm made from short synthetic threads, flock, or spun fibers [4]. The NP collection process is often reported to be uncomfortable [5]. Due to supply chain limitations and increased demand, anterior nasal (AN) specimens have been successfully adopted as an effective alternative to address NP swab shortages [6], and nasal swabs have been found to be equivalent to NP swabs for the detection of SARS-CoV-2; high sensitivity and specificity have been indicated for that sample type [7]. AN swabs offer similar testing sensitivity to NP swabs, as well as being easier to administer and more comfortable for the patients, supporting their use in large-scale screening and testing [5].

New swab production methods have been explored to address the increase in demand for nasal swabs: 3D-printed and injection-molded swabs displayed performance comparable to standard flocked swabs [4,8], while offering high-throughput, cost-effective alternatives [8,9]. In addition, a non-absorbent swab would enable the collection of samples in limited volumes and to elute them into smaller volumes of transport media, in comparison to standard absorbent swabs, resulting in greater sample concentrations and subsequent increases in sensitivity in viral RNA detection.

To validate new swabs, innovative experimental procedures are needed to streamline this process. We evaluated the performance of the novel ClearTip™ (Yukon Medical, Durham, NC, USA) injection-molded swab in comparison to traditional flocked nasal swabs. Preclinical validation was performed with an innovative in vitro tissue model to mimic the soft tissue structure as well as the viscous mucous component of the nasal passage. We hypothesized that the simulation of such tissue properties will provide a benchtop validation to further support subsequent clinical studies. Finally, we evaluated the performance of the ClearTip™ injection-molded swab in comparison to traditional flocked nasal swabs in accurately collecting patient samples as well as detecting SARS-CoV-2 in two independent clinical studies.

## 2. Materials and Methods

### 2.1. Materials

**Figure 1:**
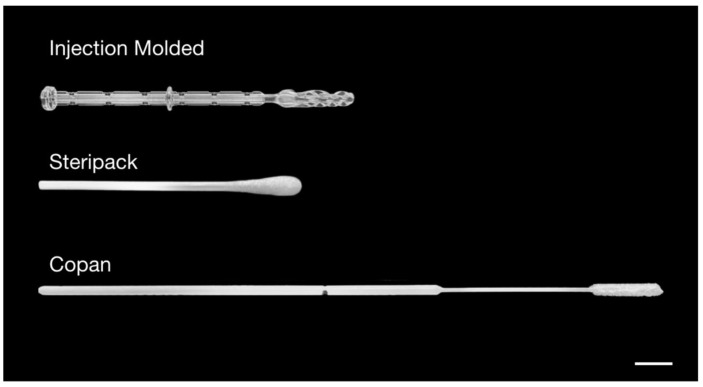
**Experimental swabs.** Macro images of ClearTip™ injection-molded, Steripack, and Copan anterior nasal swabs used in these studies. Scale bar = 10 mm.

Anterior nasal, mid-turbinate, and nasopharyngeal injection-molded swabs were designed, produced, and manufactured by Yukon Medical (Figure 1). These swabs were then compared with commercially available flocked swabs (Steripack and Copan).

### 2.2. Preclinical Studies

1. Anterior Nasal Tissue Model Preparation

A nasal tissue model was developed to perform in vitro pre-clinical studies to quantify swab pick-up and release of inactivated SARS-CoV-2 from a physiologically relevant nasal mucosa. The nasal tissue model comprised a natural sponge made of cellulose (7456T-C41, 3M, Saint Paul, MN, USA) to mimic the nature of soft tissue. It was prepared with hollow punches in a cylindrical structure with external and internal diameters of 2.5 and 0.875 mm, respectively, and overall length of 26 mm. To confine and retain the mucus, the tissue model was inserted into a polyvinyl chloride external tubing (Everbilt). The sponge was disinfected in subsequent washes of 10% bleach, 70% ethanol, and deionized water, and then autoclaved. Before mucus saturation, the model was left to dry overnight in a biological hood. The mucus mimicking solution was prepared from a 2 wt.% polyethylene oxide solution (CAS#: 25322-68-3 Acros Organics LOT#: A0403177), which has previously been shown to have similar viscosities to healthy nasal mucus [10,11]. The anterior nasal tissue model was then saturated with 4.5 mL of the physiologically relevant mucus solution.

2. Pick-Up Swab Quantification

To quantify swab uptake, the anterior nasal tissue model was saturated with the physiologically relevant mucus solution and the following swabbing procedure was utilized: each swab was inserted into the model until resistance was encountered, twisted around the nasal model surface five times, held in place for 15 s, and then removed. The pick-up swab quantification was performed by gravimetrical analysis for the ClearTip™ NP and ClearTip™ MT/N swabs in comparison to commercially available flocked swabs (Steripack), and the weight of each swab (*n* = 5) was recorded before and after the swabbing procedure and reported as the mass uptake, for three independent experiments.

3. Swab Release Quantification

To quantify swab release, the anterior nasal tissue model was saturated with the physiologically relevant mucus solution spiked at a concentration of 10^6^ copies/mL of heat-inactivated SARS-CoV-2, USA-WA1/2020 (NR-52286, BEI Resources, ATCC, USA) and the swabbing procedure in the bench model was performed, as described above. After the procedure, the swab was removed and placed in an Eppendorf tube with 350 µL of viral transport media (VTM). Immediately afterwards, the vial with the swab was vortexed for 30 s, sonicated for 1 min, and vortexed again for 30 s. Subsequently, 5 µL from each sample was then tested to quantify the detection of SARS-CoV-2, as an expression of the pick-up and release of the different swabs. To evaluate the presence of SARS-CoV-2, we utilized the CDC 2019-Novel Coronavirus (2019-nCoV) Real-Time RT-PCR Diagnostic Panel (https://www.fda.gov/media/134922/download (accessed on 8 May 2021)), as per the manufacturer’s instructions, using 2019-nCoV_N1 Combined Primer/Probe Mix with a Quantabio qScript XLT One-Step RT-qPCR ToughMix. Amplification was performed following the manufacturer’s instructions with a QuantStudio™ 5 Real-Time PCR System (Thermo Fisher Scientific, Waltham, MA, USA). The results for each swab (*n* = 5) were reported as cycle threshold (Ct) values, for three independent experiments. Following the CDC 2019-Novel Coronavirus (2019-nCoV) Real-Time RT-PCR Diagnostic Panel, a specimen is considered positive for 2019-nCoV if 2019-nCoV marker (N1, N2) cycle threshold growth curves cross the threshold line within 40.00 cycles (<40.00 Ct).

4. Preclinical Human Sampling

Laboratory staff were approached regarding volunteering for a self-sampling study. Volunteers (*n* = 8) were provided with one Copan FLOQSwabs 220250 Regular, one ClearTip™ swab, and two 15 mL conical tubes containing 3 ml of VTM. Each volunteer was asked to utilize the flocked swab in their right nasal cavity and the ClearTip™ in their left nasal cavity. Sampling for both swab types was performed identically by inserting the swab until resistance was noticed, rotated 5 times, and were then removed and placed into the separate tubes of VTM. Following swabbing, each tube of VTM was utilized to perform cell counts with the Countess II (Life Technologies, Foster City, CA, USA). Briefly, 10 µL of VTM from each tube was mixed with 10 µL Trypan Blue (Life Technologies), and 10 µL of the mixture was loaded onto a Countess II slide. Cell counts were performed utilizing the default Countess II settings. Following cell counting, 200 µL of VTM from each tube was extracted for RT-qPCR analysis, utilizing the Nuclisense EasyMag (Biomeriuex, Durham, NC, USA), following the manufacturer’s instructions. Post-extraction, each sample was analyzed for total DNA concentration, utilizing the NanoDrop One (Thermofisher Scientific, Waltham, MA, USA), as per the manufacturer’s instructions. Samples were subsequently analyzed for RNase P, utilizing the CDC nCOV_2019 RT-qPCR assay, as per the manufacturer’s instructions. Cell counts, total DNA concentration, and RNase P Ct values were utilized to discern differences in the quantity of cells released by each swab type.

### 2.3. Clinical Studies

To evaluate ClearTip™ versus flocked swab performance, two clinical studies were carried out to assess the pick-up and release of each swab type by quantifying human RNase P expression, as well as N1 and N2 when the subjects were positive.

#### 2.3.1. Clinical Study I

1. Study design and Oversight

Participants were already enrolled into a surveillance program at the University of Massachusetts Lowell (UML), and were asked to be swabbed with the ClearTip™ N swab as well as with a commercially available comparator (Steripack), and to complete a short survey on the comfort of the swabs. Swabs were collected in VTM in a 15 mL conical tube, transported to the RADx Validation Core Lab at UML, stored at 4 °C identically to clinical samples, and tested the following day. Sample collection was performed by trained nursing personnel at the UML Wellness Center. This study was reviewed and approved by the Institutional Review Board of UML (protocol number 20-149-BUC-FUL). Due to Institutional Review Board limitations, we cannot provide identifiable demographic information for the enrolled participants.

2. Participants

Participants were healthy individuals, as self-reported and determined by screening performed during recruitment consent, and already enrolled into the surveillance program at UML. Patient AN samples (*n* = 47) were collected with a ClearTip™ swab within seven days of a validated negative SARS-CoV-2 test result from a sample collected with a CLIA-approved Class I-exempt nasal swab and tested with an approved RT-qPCR assay by a CLIA-certified clinical lab. Adults over 18 years of age were given a participant information sheet by study staff and asked whether they would agree to being swabbed with an experimental swab by a trained nurse for the control swab required for testing, and also complete a short comfort survey.

3. Study procedures

ClearTip™ injection-molded swabs were individually packaged and autoclaved for sterilization, according to the manufacturer’s protocols. Swabbing was performed as per the standard protocol in both nostrils for each swab. After swabbing both nostrils, the subject returned to the study coordinator(s). Participants then completed a short survey on the relative comfort of the two swabs. They rated each swab on a 5-point Likert scale, with 1 = very uncomfortable and 5 = very comfortable. The order of swabbing with the control or the experimental swabs was random for all the participants. Control and experimental swabs were placed in separate vials of VTM and transported to the RADx Validation Core Lab, where each sample was tested on the QuantStudio™ 5 Real-Time PCR System with 2019-nCoV_N1, 2019-nCoV_N2 and Human RNase P Combined Primer/Probe Mix with a Quantabio qScript XLT One-Step RT-qPCR ToughMix, as per the standard CDC protocol.

#### 2.3.2. Clinical Study II

1. Study Design and Oversight

Participants, enrolled in the study performed at Washington University School of Medicine, were recruited as symptomatic at a walk-in clinic. They were all tested first with NP swabs and then randomly swabbed nasally first with the ClearTip™ N swab or a commercially available comparator (Copan). Swabs were eluted for 10 min in M4-RT universal transporting medium (UTM). Sample collection was performed by trained nursing personnel at the Washington University School of Medicine. This study was reviewed and approved by the Institutional Review Board of Washington University School of Medicine (protocol number 7453). Due to Institutional Review Board limitations, we cannot provide identifiable demographic information for the enrolled participants.

2. Participants

Participants were symptomatic individuals, as self-reported and determined by screening performed during recruitment consent, with a confirmed EUA-approved RT-PCR COVID-19 virus test result, performed with nasopharyngeal (NP) flocked swabs processed with Cepheid Xpert^®^ Xpress SARS-CoC-2. Patient AN samples (*n* = 38) were collected with a ClearTip™ swab in comparison to flocked swabs (FLOQSwabs™ Contoured Adult, Copan). Adults over 18 years of age were given an informed consent form by study staff and asked whether they would agree to being swabbed with an experimental swab by a trained nurse to the control swab required for testing.

3. Study procedures

ClearTip™ swabs were individually packaged and autoclaved for sterilization according to manufacturer protocols. Two paired anterior nares nasal swabs from each nare were collected from each enrolled subject. After swabbing both nostrils, the subject returned to the study coordinator(s). The order of swabbing with the control or the experimental swabs was random for all the participants. Control and experimental swabs were placed into the M4-RT UTM and shipped to shipped to the Microbiology Lab at the Grady Memorial Hospital (Atlanta, GA, USA), stored at 4 °C, identically to clinical samples, and tested the following day. Samples were tested on the QuantStudio™ 5 Real-Time PCR System with 2019-nCoV_N1, 2019-nCoV_N2 and Human RNase P Combined Primer/Probe Mix with a Quantabio qScript XLT One-Step RT-qPCR ToughMix, as per the standard CDC protocol.

### 2.4. Statistical Analyses

Paired *t*-tests and equivalence testing using a two one-sided *t*-test (TOST) of equivalence using appropriate equivalence margins were performed with a *p* < 0.05 significance threshold [12]. Negative predictive values (NPVs) were calculated with a weighted generalized score method [13]. Paired *t*-tests, equivalence tests and the weighted generalized score method were analyzed using SAS 9.4 (SAS Institute, Inc., Cary, NC, USA). Additional Student’s *t*-tests (*t*-tests) with a *p*-value < 0.05 were performed with Origin (Pro), Version 2021b OriginLab Corporation, Northampton, MA, USA.

## 3. Results

### 3.1. Benchtop Validation

**Figure 2:**
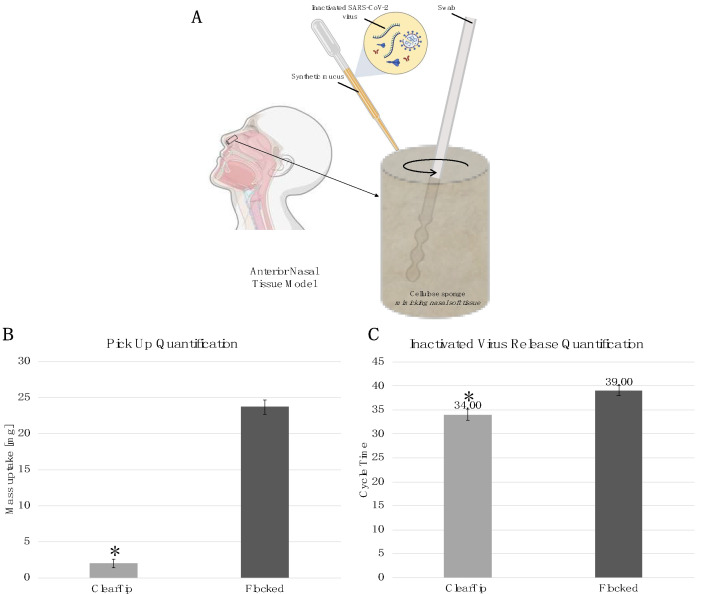
**ClearTip^TM^ benchtop validation.** (**A**) Anterior nasal tissue model comprising a cellulose sponge loaded with synthetic nasal mucus spiked with inactivated SARS-CoV-2. The cellulose sponge mimicked the nasal soft tissue during the benchtop swabbing procedure with the ClearTip^TM^ in comparison to flocked control swab. (**B**) Mass uptake during the benchtop swabbing procedure was quantified by gravimetrical analysis. Flocked control samples displayed a statistically greater mass update compared with ClearTip^TM^. (**C**) Swab material release was collected into viral transport media, and inactivated virus was quantified using PCR following CDC guidelines. * Statistical difference against flocked control group (*p* < 0.05). Created with BioRender.com (Accessed on 9 January 2021).

An anterior nasal tissue model was developed to quantify the sample uptake and release from anterior nasal swabs (Figure 2A). The tissue model was designed with a cellulose sponge to mimic the soft nasal tissue, and saturated with synthetic nasal mucus spiked with inactivated SARS-CoV-2; this was designed to mimic a clinical nasal swab to facilitate an evaluation of swab performance. The swab uptake was quantified by gravimetric analysis after the benchtop swabbing procedure. The ClearTip™ swab displayed a significantly lower uptake of synthetic mucus in comparison to control flocked swabs (*p* < 0.05). ClearTip™ demonstrated a more than 20-fold reduction in swab uptake in comparison to the control swab (Figure 2B). The ability of the swabs to release biological materials was quantified by performing the swabbing workflow with the nasal tissue model loaded with inactivated SARS-CoV-2. Swabs were then collected into viral transport media, and the virus released from the swab was quantified via RT-qPCR, in accordance with CDC guidelines. ClearTip™ displayed a significantly lower cycle time (e.g., more virus) in detecting inactivated SARS-CoV-2, in comparison to the flocked control swab (*p* < 0.05). The reduction in cycle time for the ClearTip™ swab supports greater viral recovery in comparison to the flocked control (Figure 2C).

### 3.2. Preclinical Human Sampling

**Figure 3:**
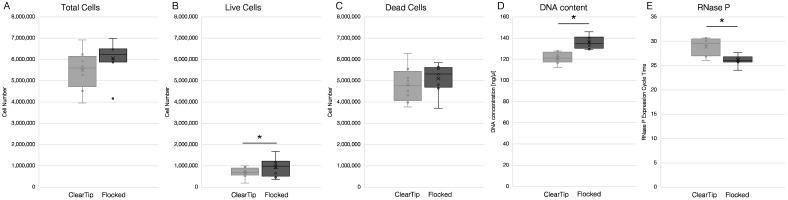
**ClearTip^TM^ Preclinical self-sampling validation.** Pre-clinical self-sampling study was performed to compare performance of ClearTip^TM^ swab against flocked swab. In this study, numbers of total cells (**A**), live cells (**B**), and dead cells (**C**) were quantified, as well as DNA content (**D**) and RNase P expression (**E**). * Statistically significant differences were only reported for live cells, DNA content and RNase P (*p* < 0.05).

Results from the preclinical self-sampling study are summarized in Figure 3. Numbers of total cells, live cells and dead cells were quantified, as well as the quantification of DNA content, and RNase P expression. Overall, samples that were from self-sampling with flocked swabs produced higher live cell counts (Figure 3B), greater DNA concentration (Figure 3D), and lower RNase P Ct values (Figure 3E) than the samples from ClearTip™ self-sampling (*p* < 0.05). Quantification of the total numbers of cells (Figure 3A) and dead cells (Figure 3C) were not statistically significantly different at the 0.05 level. Although the *t*-test showed non-significant differences (*p* = 0.0509 and *p* = 0.2920 for total and dead cells, respectively), the underpowered sample size and large variation contributed to the non-equivalence result in the equivalence test.

### 3.3. Clinical Studies

**Figure 4:**
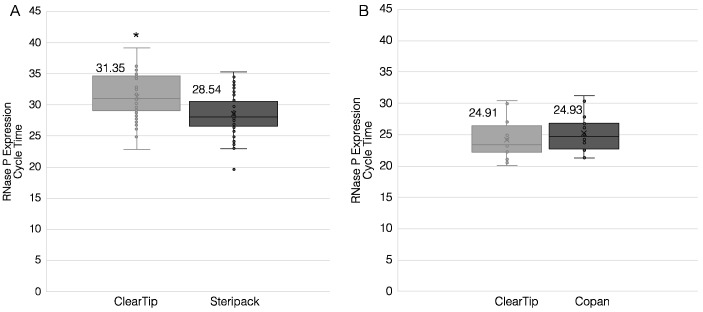
**ClearTip^TM^ clinical validation for RNase P detection.** Two independent clinical studies were performed to quantify the ability of the ClearTip^TM^ swab to pick up nasal cellular material in comparison to control flocked nasal swabs. (**A**) Clinical study at the CAPCaT University of Massachusetts Lowell showed a significant increase in cycle time for the detection of RNase P collected by ClearTip^TM^ Swabs and Steripack Nasal Swabs (*p* < 0.05). (**B**) Clinical study at Washington University St. Louis showed no statistical difference (*p* = 0.945) in cycle time for the detection of RNase P collected by ClearTip^TM^ Swabs and Copan Nasal Swabs. * Statistical difference against flocked NP control (*p* < 0.05).

To quantitively compare the performance of the ClearTip™, 47 participants self-collected nasal samples using ClearTip™ in comparison to a flocked CLIA-approved swab (Figure 4A). None of the participants tested positive in this surveillance program; thus, the expression of RNase P was used to compare the efficiency of each swab to pick up human biological material. ClearTip™ demonstrated a significantly higher RT-qPCR cycle time in comparison to the flocked control swab, Steripack (*p* < 0.05). Quantitatively, we reported a two-cycle difference for the two compared swabs, which was found to be statistically different.

**Figure 5:**
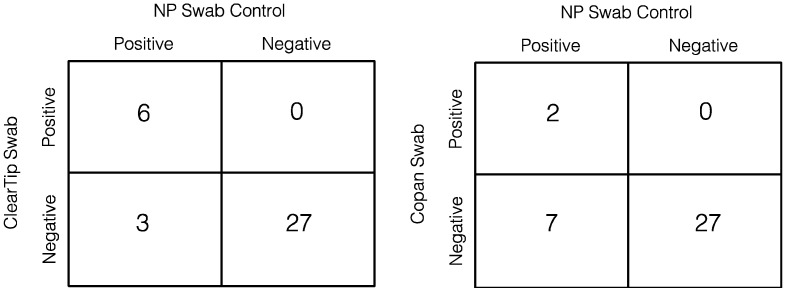
**ClearTip^TM^ concordance with control swabs.** Summary of positive and negative samples detected with the ClearTip™ swab in comparison to Copan flocked swab. The statuses of participants enrolled in the study were confirmed by an EUA-approved RT-PCR COVID-19 virus test result, performed with NP flocked swabs.

In the second clinical study, 38 participants were sampled using ClearTip™ in comparison to a flocked CLIA-approved swab (Figure 4B). ClearTip™ displayed no differences in comparison to the CLIA-approved swab, Copan (*p* = 0.9549). In addition, an equivalence test with a margin of 1 determined that the swabs measured the same value of RNase P. Using an RNase P RT-PCR, there was no difference between ClearTip™ and the Copan swabs. Of the 9 individuals (of 38 in total) who tested NP-swab-positive in a CLIA-certified reference lab (Figure 5), the ClearTip™ swab confirmed 78% of positive cases detected with an NP CLIA-approved flocked swab for N gene detection, whereas the Copan swab confirmed only 22% of the cases. Based on a weighted generalized score method, ClearTip™ displayed an NPV of 90%, whereas the comparator swabs showed an NPV of 79% (*p* < 0.05).

**Figure 6:**
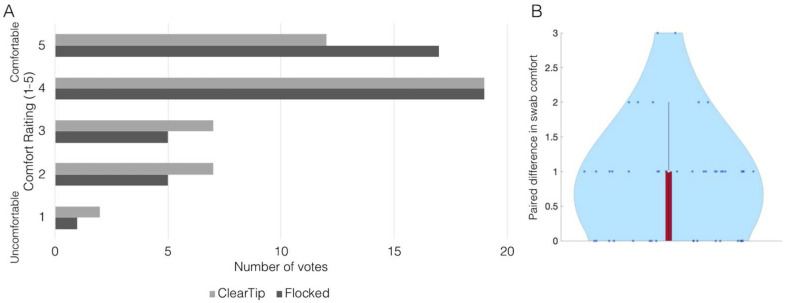
**ClearTip^TM^ comfort study.** (**A**) Summary of comfort survey with ratings on a 5-point Likert scale, with 1 = very uncomfortable and 5 = very comfortable. (**B**) Mean paired difference in swab comfort. ClearTip™ was found to be equivalent to the flocked CLIA-approved swab with a paired *t*-test equivalence margin of 1 (*p* < 0.05).

In the first clinical study, participants scored the comfort of the two swabs on a 5-point Likert scale (Figure 6A). The mean paired difference in swab comfort was reported as 0.3 (Figure 6B). ClearTip™ was found to be equivalent to the flocked CLIA-approved swab with a paired two one-sided *t*-test (TOST) of equivalence using an equivalence margin of 1 (*p* < 0.05).

## 4. Discussion

During the peaks in the pandemic, swab shortages at hospital across the United States and worldwide have been reported [14], prompting the need to develop new swab prototypes with comparable performance and capability for high-volume manufacturing, while being cost-efficient. Here, we report a new injection-molded anterior nasal swab, ClearTip™, that was experimentally validated in a laboratory setting as well as in independent clinical studies in comparison to gold standard flocked swabs.

In vitro tissue models have recently been developed and extensively investigated because they can mimic a wide range of physical, structural, and biological characteristics of native tissues, and have thus been implemented to study in vitro physiological and pathological tissue conditions [15,16,17,18,19].

We report an anterior nasal tissue model which mimics the architecture and structure of the native soft tissue, as well as the physical properties of the nasal mucus. In the anterior nasal tissue model, we quantified the ability of ClearTip™ to pick up artificial mucus in comparison to gold standard flocked swabs. ClearTip™ displayed more than 20 times less retention than a flocked swab. However, when we evaluated the capacity of ClearTip™ to release viral material in vitro, the injection-molded swab displayed significantly greater inactivated virus release in comparison to flocked swabs. This suggests a much greater release efficiency of inactivated virus from the ClearTip™ swab, which may offset the reduction in uptake in comparison to the standard flocked swab.

In clinical assessments, ClearTip™ exhibited variability in its ability to pick up and release cellular material as a proxy for performance in COVID-19 testing. Measurements of cell population, DNA concentration, and RNase P quantification showed equivalence in one study and a reduction in performance in comparison to flocked swabs in a separate assessment. This suggests that the protocol for use may make a difference in the ability of injection-molded swabs to pick up cells and, subsequently, viruses. Therefore, future sampling protocols for injection-molded swabs may benefit from including vertical nasal wall scraping, in addition to a circular motion collection mode, to reduce interpersonal variability. In a small clinical assessment, flocked and ClearTip™ nasal swabbing detected 22% and 78% of NP swab SARS-CoV-2-positive paired samples, respectively, supporting further future studies.

The in vitro anterior nasal tissue model offers a novel, efficient, and clinically relevant validation tool that allows replication of the clinical swabbing workflow with high fidelity, while being accessible to researchers, safe, reproducible, and time- and cost-effective. We also demonstrated the concordance of the in vitro model results with our clinical studies. The non-absorbent injection-molded ClearTip™ swab could have the potential to overcome future swab shortages, due to its manufacturing advantages, while offering benefits in comparison to highly absorbent swabs in terms of comfort, limited volume collection, and potential multiple usage.

Injection-molded swabs can be mass produced at limited costs from well-characterized polymeric materials, while leveraging years of experience in medical devices manufacturing from injection molding processes. In addition, the non-absorbent head and superior material release may allow greater sensitivity when used in pooling studies, although the less efficient capture of cells with the current instructions for use may require optimization of the technique. The single solid material design could potentially allow multiple use of the same injection-molded swab, after cleaning, disinfection, and sterilization, under extenuating circumstances, such as those several low- and middle-income countries have faced in the midst of the COVID-19 pandemic.

## Data Availability

The data that support the findings of this study are available from the corresponding author, C.E.G., upon reasonable request.
